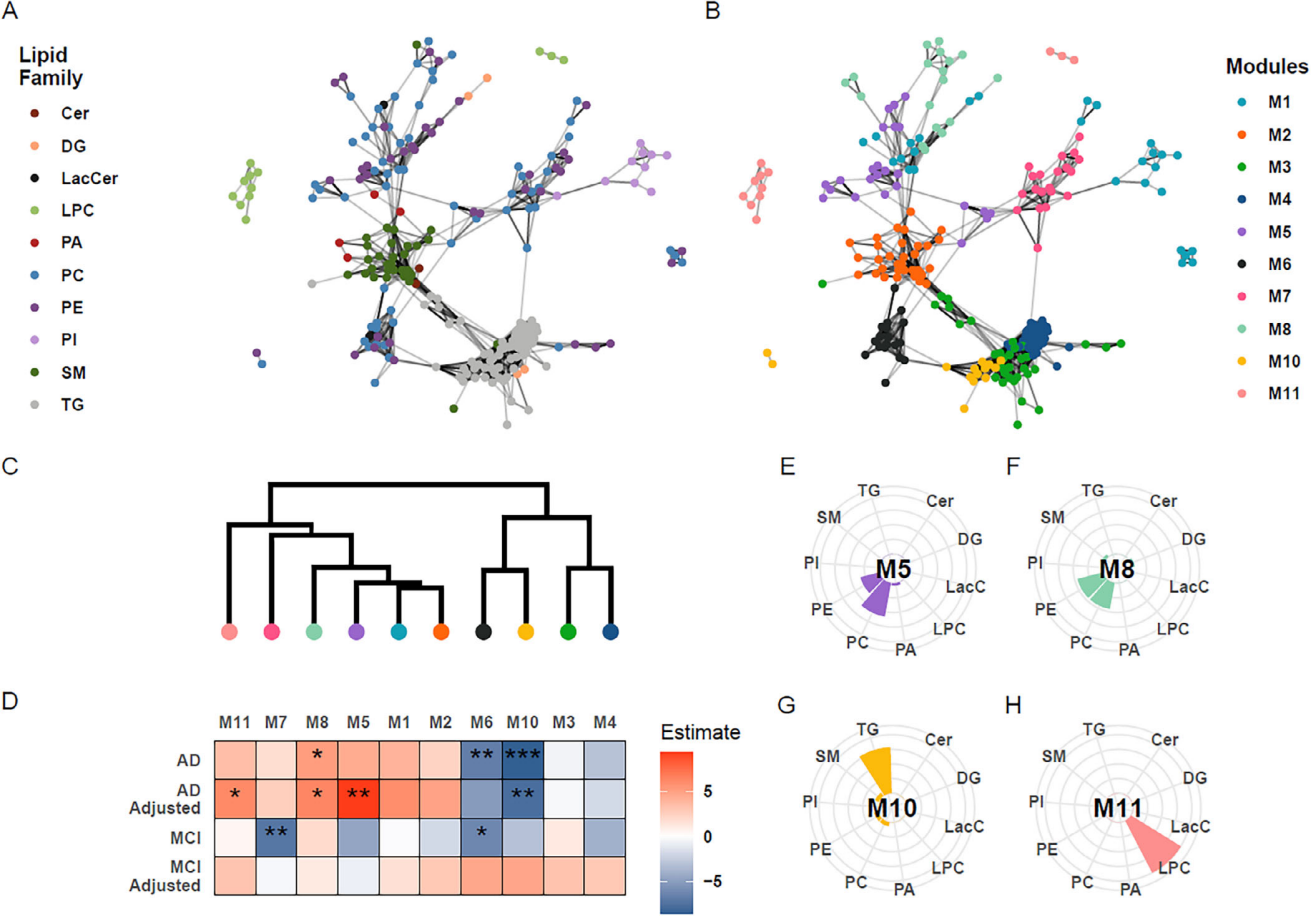# Comprehensive lipidomic analysis for identification of lipid biomarkers associated with Alzheimer´s disease

**DOI:** 10.1002/alz.089130

**Published:** 2025-01-09

**Authors:** Asger Wretlind, Jin Xu, Petroula Proitsi, Cristina Legido‐Quigley

**Affiliations:** ^1^ Institute of Psychiatry, Psychology and Neuroscience, Maurice Wohl Clinical Neuroscience Institute, King’s College London, London UK; ^2^ Centre for Preventive Neurology, Wolfson Institute of Population Health, Queen Mary University of London, London UK; ^3^ Steno Diabetes Center Copenhagen, Copenhagen Denmark

## Abstract

**Background:**

Alzheimer’s disease (AD) is a devastating disease at an individual level and for the wider society. Despite huge research efforts the underlaying causes of AD is still not well understood. We know that lipid metabolism is fundamental for maintaining a heathy brain and that some of the strongest risk factors for AD, such as APOE4, affect lipids. In this study we set out to measure the lipid changes associated with AD.

**Method:**

We used a total of 841 participants from the AddNeuroMed and Dementia Case Register cohorts: 306 people with AD, 165 people with mild cognitive impairment (MCI) (n = 165), and 370 healthy controls.

Mass spectrometry based lipidomics was carried out on plasma samples using a Waters ACQUITY UPLC and XEVO QTOF‐MS.

Lipid molecules were clustered into modules based on correlation networks using Weighted Gene Correlation Network Analysis (WGCNA). Module eigengenes were used to investigate the association of each module with MCI and AD. Key lipid drivers (‘Hub’) associated with AD were selected and investigated further. Linear regression analyses were used to investigate the association of modules and key lipids with AD and MCI status after adjustment for sampling site, age, sex and presence of APOE4 allele. The False Discovery Rate (FDR) was applied to control for multiple testing.

**Result:**

Lipidomics analysis resulted in 261 identified lipid molecules, which were analyzed with WGCNA resulting in 10 robust modules. Four modules were significantly associated with AD vs. healthy control after adjusting for covariates and following correction for multiple testing. Investigating the lipid drivers of these modules highlighted 55 lipid hubs, 26 of which were significantly associated with AD vs. control, after adjustment for confounders and multiple testing. These lipids included triglycerides, phosphatidylcholines, phosphatidylethanolamines and lysophosphatidylcholines.

**Conclusion:**

Here, we present a workflow for robust identification of lipid molecules associated with Alzheimer’s disease. Further work will establish how these lipids relate to AD risk factors, clinical measures, and genetic risk for AD.